# How does the air pollution prevention and control action plan affect sulfur dioxide intensity in China?

**DOI:** 10.3389/fpubh.2023.1119710

**Published:** 2023-01-26

**Authors:** Shuhai Niu, Yidong Chen, Ruiwen Zhang, Yanchao Feng

**Affiliations:** Business School, Zhengzhou University, Zhengzhou, China

**Keywords:** sulfur dioxide intensity, air pollution control policy, total factor productivity, quasi-natural experiment, China

## Abstract

As a part of China's efforts to mitigate and control air pollution in key areas, the Air Pollution Prevention and Control Action Plan was implemented in 2013, and several regulatory measures were introduced. Based on the data from 271 prefecture-level cities between 2008 and 2018, the difference-in-differences model is used to explore the effect of it on sulfur dioxide intensity in our study, and several significant results are as follows: (1) The baseline results suggest a 23% reduction in sulfur dioxide intensity in pilot cities compared to non-pilot cities. (2) The total factor productivity fails to play a partial mediating role in reducing the sulfur dioxide intensity under the implementation of the policy. (3) The results of the triple differences model suggest that the policy still exerts significant adverse effects on sulfur dioxide intensity in the pilot areas of the carbon emission trading scheme.

## 1. Introduction

**Figure 1:**
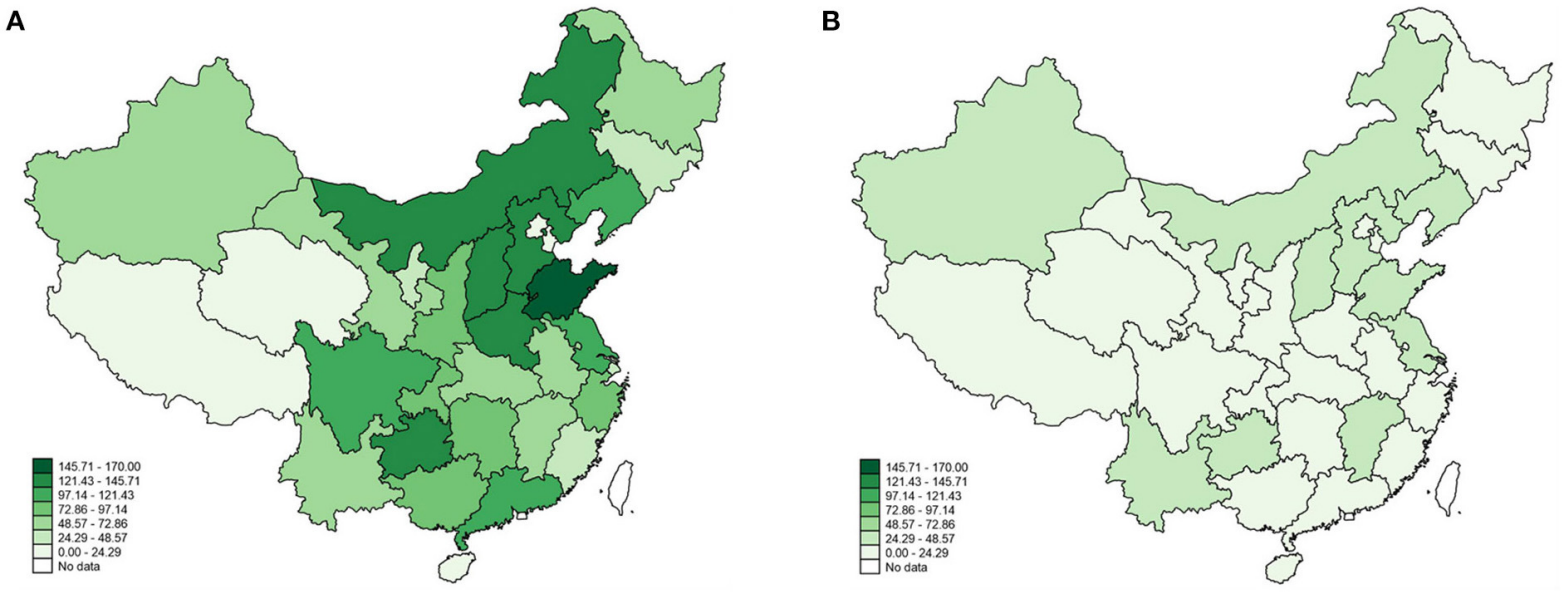
Sulfur dioxide emission in **(A)** 2008 and **(B)** 2018.

Taking advantage of China's reform and openness, China has made remarkable economic progress and improved people's living conditions. However, it also leads to some problematic environmental issues. Air pollution, represented by sulfur dioxide pollution, is a serious problem that many developing countries, such as China, pay close attention to Arceo et al. (1). On the one hand, sulfur dioxide pollution causes very high environmental costs due to its contribution to fog-haze episodes (2). However, sulfur dioxide pollution can adversely affect human health and ecological environment (3). China's air pollution is severe as the world's largest energy consumer (4). For example, the 2007 World Development Indicators data show that some cities exceed the maximum air pollution index (above 500), while more than 60% of the world's most polluted cities are located on the Chinese mainland. According to the 2018 Environmental Performance Index (EPI), China's environmental performance index ranked fourth from the bottom in 2018. Although the Chinese government has implemented a series of policies since the 1980's to control sulfur dioxide emissions and improve air quality, sulfur dioxide pollution remains high due to large amounts of sulfur dioxide emissions into the atmosphere in some regions of China (5). From the comparison presented in Figure 1, areas polluted with sulfur dioxide are still primarily concentrated in the central and eastern regions. However, sulfur dioxide emissions in 2018 have decreased, compared to sulfur dioxide pollution in 2008.

**Figure 2:**
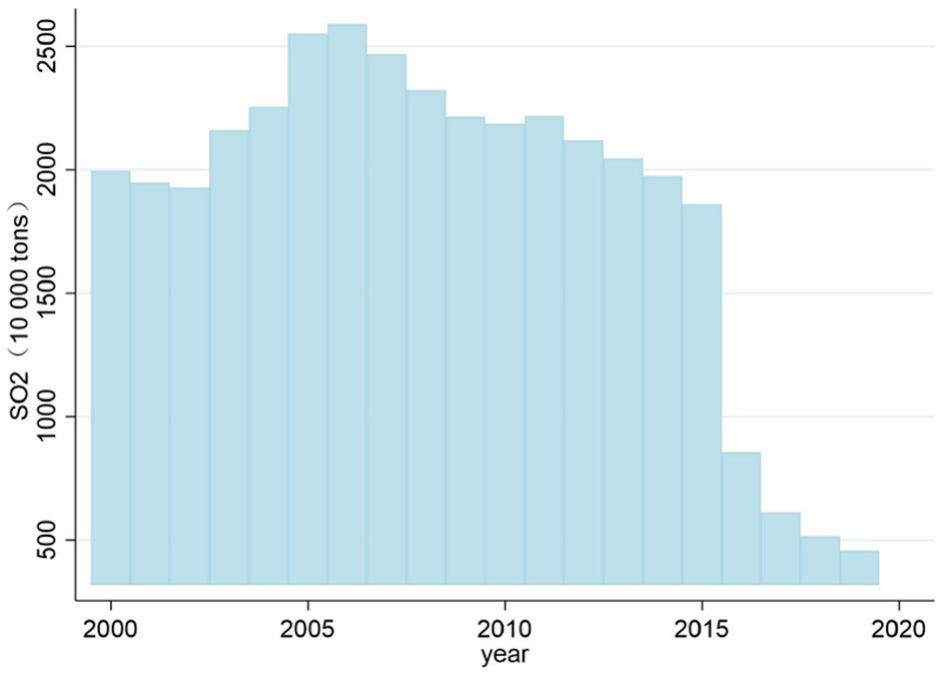
National sulfur dioxide emission changes from 2000 to 2020.

**Figure 3:**
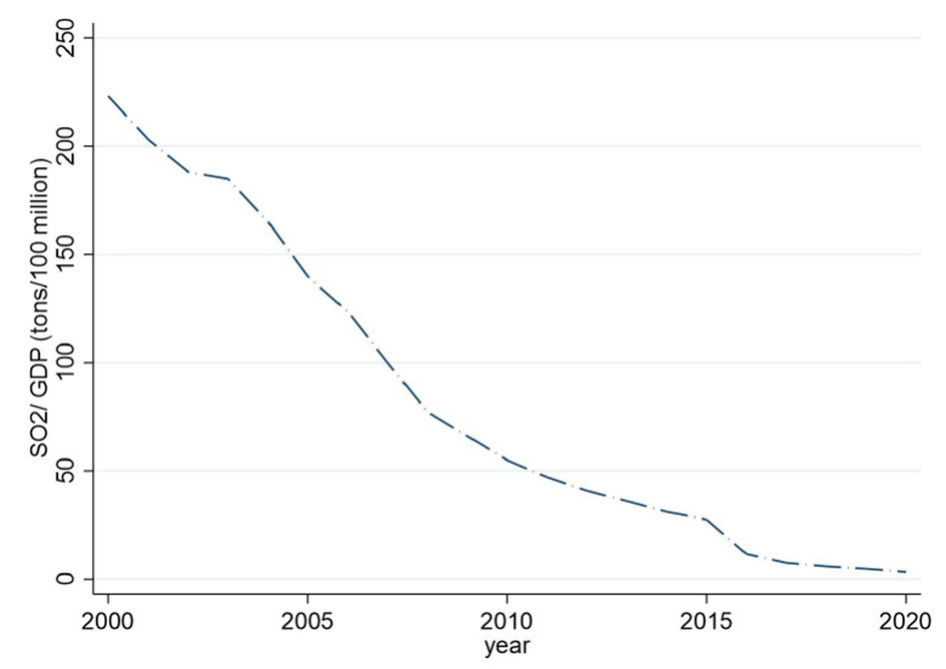
National sulfur dioxide intensity changes from 2000 to 2020.

To improve air quality, China has been doing its best to control air pollution, as evidenced by the 148 policy documents issued by the central government from 1973 to 2016 (6). For instance, the first national conference on environmental protection was held in 1973, marking the beginning of China's air pollution control efforts. In addition, the Tentative Standards for Industrial Wastes Emission was promulgated in 1973, which set industrial emission standards. In 1995, China tried to achieve coordinated development of the regional economy, society and atmospheric environment by issuing air pollution regulatory standards, formulating planning opinions, issuing action plans, and implementing policies and measures. In 2000, the Chinese government promulgated the Law on the Prevention and Control of Air Pollution, aiming to establish “two control areas” throughout the country, namely acid rain prevention and control areas and sulfur dioxide prevention and control areas. However, implementing this policy can only curb the trend of acid rain and sulfur dioxide pollution. It cannot reverse the problem of acid rain and sulfur dioxide pollution. In particular, since General Secretary Xi Jinping took office, the country has paid more attention to controlling air pollution. In 2013, the State Council issued the “Action Plan for Prevention and Control of Air Pollution,” which contains 10 provisions requiring that by 2017, the PM_10_ concentration at the prefecture level and above cities will drop by more than 10% compared to 2012, and the number of days with air quality standards will increase year by year. PM_2.5_ concentrations in the Beijing-Tianjin Hebei region, the Yangtze River Delta, and the Pearl River Delta should fall by about 25, 20, and 15%, respectively (7). In addition, the APPCAP policy has been called the strictest air pollution control system in human history. From the data in Figures 2, 3, we can see that in the second year after the implementation of the APPCAP policy, sulfur dioxide emissions decreased significantly in 2015. At the same time, the sulfur dioxide intensity also maintained a downward trend year by year, but it remains to be explored whether this benefit from implementing the APPCAP policy is worth further exploration.

Although there is growing evidence of the correlation between environmental protection policy and environmental pollution (7–9), there is a massive scarcity of empirical effort regarding China's APPCAP policy on air pollution intensity, especially sulfur dioxide intensity. We regard the implementation of the APPCAP policy in China as a quasi-natural experiment and study the impact of the implementation of the APPCAP policy on sulfur dioxide intensity. In 2013, the Chinese government established the APPCAP pilot region in 47 cities. The APPCAP policy not only strictly constrains the total amount of pollutants discharged, but also emphasizes the comprehensive treatment of sulfur dioxide, NO compounds, volatile organic compounds and micro-particulate matter. The APPCAP policy can guide more social capital in the field of air control and restrict the production activities of the heavily polluting industry to a certain extent, thus benefiting air pollution control. In this context, concerns are raised: Can the APPCAP policy reduce the intensity of sulfur dioxide? If so, through which channels? Also, is there heterogeneity in the APPCAP policy effects on sulfur dioxide intensity among different regions? Answering these issues provides helpful insights for China and other developing countries to formulate similar environmental governance policies.

The contributions of our paper are primarily manifested in four aspects. First, our research differs from previous literature on the effect of air pollution control. We focus on pollution on the intensity level, which provides a novel perspective for analyzing the air pollution control effect of APPCAP. Secondly, in this paper, various models are applied, such as, DID model, PSM-DID model, IV test, DDD model, etc., which effectively solve the problems of model selection bias, endogenous problem, and sample selection bias and obtain sound research conclusions. Third, to clarify the impact mechanism and mediating influence, we select total factor productivity (TFP) as the mediating variable in this paper. Last but not least, exploring the sulfur dioxide intensity reduction effect of the APPCAP policy can provide strong evidence for further expansion of this plan in China and benefit other developing countries seeking to reduce air pollution.

The remainder of our paper is structured as follows: Section 2 presents the literature review, and the empirical strategy is described in Section 3. Section 4 shows the empirical results and a series of analyses. Section 5 draws conclusions and policy recommendations.

## 2. Literature review

Our paper is mainly related to three types of literature. The first type of literature is on research on energy efficiency and air pollution. Energy efficiency is divided into single-factor energy efficiency and total-factor energy efficiency. Although single-factor energy efficiency can be measured in terms of energy consumption per capita of GDP, total-factor energy efficiency considers the impact of multiple input-output variables on energy factors. Gu and Yan (10) used data from all A-share listed companies from 2008 to 2018 to find that the promulgation of ambient air quality standards can significantly reduce PM_2.5_ concentrations, and its implementation has remarkably improved the total factor productivity of listed enterprises in pilot cities. Wang et al. (11) found that regulating the atmospheric environment can significantly inhibit the growth of industrial total factor productivity by selecting panel data from 37 industrial sectors in China from 2003 to 2016 as the research object. Using the panel vector auto-regressive method (PVAR) and panel data from 30 provinces in China between 2005 and 2018, Li et al. (12) found that improving agricultural green total factor productivity by significantly reducing air pollution, in line with the work of Ahmed et al. (13), who used the PMG method and panel data of 50 states of America from 2005 to 2019. Based on the data from 30 provinces on the China from 2004 to 2017, Liu et al. (14) use the difference-in-differences (DID) method to find that the APPCAP policy has promoted the improvement of provincial energy efficiency. However, the impact on energy efficiency in different provinces is heterogeneous. Referring to the research ideas and methods of the above paper, it is necessary to consider the total factor productivity in our study and explore the role of the total factor productivity in the control of air pollution by the APPCAP policy.

The second type of literature describes and estimates the temporal and spatial changes in air pollutants after implementing the APPCAP policy. Based on panel data from 271 prefecture-level cities from 2008 to 2018 and using difference-in-differences (DID) and propensity score matching difference-in-differences (PSM DID) methods, Yu et al. (7) found that APPCAP significantly reduced PM_2.5_ concentration and SO_2_ emissions in the pilot areas. Based on the panel data of PM_2.5_ concentration in 197 cities at the prefecture level and above in China from 2006 to 2016, Zhao et al. (15) found that the APPCAP policy has a significant smog control effect on the Beijing-Tianjin-Hebei region, but do not have noticeable policy effects on the Yangtze River Delta and Pearl River Delta regions. Zhang et al. (16) found that APPCAP effectively reduced China's per capita carbon emissions at the national level without considering spatial spillover effects. Using the empirical data of 109 resource-based cities from 2004 to 2018, Wu et al. (17) find that air pollution prevention and control actions significantly affect the air quality of growing, mature, and declining cities. However, the policy effect on regenerative resource-based cities is not apparent. However, Huang et al. (18) estimated the health impact of APPCAP on 74 critical cities from 2013 to 2017, suggesting that the effect of APPCAP on ozone and nitrogen dioxide emission control was not noticeable.

The third type of literature is the impact on human physical and mental health and disease risk after implementing the APPCAP policy. For example, Maji et al. (19) used air pollution data from 35 points in Beijing from 2014 to 2018 and found that Beijing's air quality improved significantly over the past 5 years, while PM_2.5_ and O_3_ mortality rates dropped remarkably. Yue et al. (20) combine chemical transport models with remote sensing and monitoring data and find a reduction in disease and mortality associated with exposure to lower concentrations of PM_2.5_ after implementing APPCAP. Zhao and Kim (21) use nationally representative CFPS survey data conducted in 2012, 2014, and 2016 and find that APPCAP significantly reduces physician-diagnosed chronic diseases of the respiratory and circulatory systems. However, Yue et al. (20) found that the policy implementation effect of APPCAP is limited. More ambitious policies are needed if DAPP (deaths attributable to PM_2.5_ pollution) is to be further significantly reduced.

In summary, although the existing literature has done much affluent research on the implementation effect of the APPCAP policy from different perspectives, there is still a lack of analysis of the national distribution data of APPCAP pilot cities (7), as well as little if any empirical work has been done to investigate the APPCAP policy effect on sulfur dioxide intensity.

## 3. Empirical strategy

### 3.1. Empirical framework

Taking China's APPCAP policy as a quasi-natural examination, we conduct a DID model to explore such policy's impact on sulfur dioxide intensity. As a magnificent tool for policy assessment, the DID estimation can principally avoid endogeneity problems. Explicitly, our DID estimation can contrast sulfur dioxide intensity in APPCAP cities (treatment group) and non-APPCAP cities (control group) in the pre-and post-implementation of the APPCAP policy, and the estimated equation is as follows.


(1) $$\begin{array}{l} ISO_{it}=\alpha_{0}+\alpha_{1}Time_{i}\times Treat_{t}+\gamma_{i}\;Control_{it}+\mu_{i}+\theta_{i}+\varepsilon_{it} \end{array}$$

Where *i* denotes city, *t* indexes year, respectively. *ISO*_*it*_ is the independent variable and refers to sulfur dioxide intensity. If city *i* implements the APPCAP policy, the value of *Time*_*i*_ is 1, and 0 otherwise. *Treat*_*i*_ is the dummy variable. *Treat*_*i*_ =1 denotes the APPCAP policy has been implemented and *Treat*_*i*_ = 0 represents the APPCAP policy has not been implemented. *Control*_*it*_ is a series of control variables. The city fixed effects (μ_*i*_), and the year fixed effects (θ_*t*_) are also included in our equation. ε_*it*_ indicates the random error term.

The coefficient of *Time*×*Treat*, α_1_, captures the net impact of China's APPCAP policy on sulfur dioxide intensity. Explicitly, a negative and significant indicates that the APPCAP policy effectively reduces sulfur dioxide intensity and approves the pollution-reduction effect of the APPCAP policy. Instead, a positive and significant indicates that the APPCAP policy aggravates sulfur dioxide intensity. Furthermore, the insignificant suggests that the APPCAP policy fails to influence pollutant emissions. In addition, *Control*_*it*_ implies the control variables selected in this study, including per capita GDP, industrial structure, industrialization level, population density, FDI, temperature, and rainfall.

According to the method of Wen et al. (22), in order to figure out the mediating effect of the mechanism, we choose the total factor productivity (TFP) as our mediating variable to investigate the extent of the influence of the explanatory variable on the explained variable through the mediating variable. Furthermore, the explicit test model is as follows:


(2) $$\begin{array}{lll} ISO_{it} & = & \beta_{1}+\omega_{1}Time_{i}\times Treat_{t}+\rho_{1} \end{array}$$

(3) $$\begin{array}{lll} TFP_{it} & = & \beta_{2}+\omega_{2}Time_{i}\times Treat_{t}+\rho_{2} \end{array}$$

(4) $$\begin{array}{lll} ISO_{it} & = & \beta_{3}+\omega_{3}Time_{i}\times Treat_{t}+\omega_{4}TFP_{it}+\rho_{3} \end{array}$$

Where β represents a constant and ω represents a coefficient Matrix, ρ represents the error term, and *TFP*_*it*_represents the total factor productivity. In addition to using the OLS estimation method, the Bootstrap test is also used to verify whether TFP played an intermediary role in the APPCAP policy's effect on sulfur dioxide intensity in our study. Among them, the Bootstrap method is a non-parametric estimation method. When the final Bootstrap confidence interval does not contain a value of 0, the mediating effect is significantly not equal to 0. Through the Bootstrap test, the robustness of the mediation effect can be further judged.

The test step is as follows: In step (1), we test the total effect; if the coefficient ω_1_ is significant, step (2) is performed; otherwise, the test ends. In step (2), we test the coefficients ω_2_ and ω_4_, and when both are significant, the indirect effect is significant, and we go directly to step (4) for further testing. If at least one of the two is not significant, the test for step (3) will be performed. In step (3), we perform a bootstrap check. When the results are significant, indicating significant indirect effects and the procedure progresses to step (4); Otherwise, the mediating effect is insignificant, and the test stops. In step (4), we test the coefficient ω_3_; if the result is significant, it indicates that the direct effect is significant, and the process enters step (5); if it is not significant, then the direct effect is not significant, we only find an intermediate effect, and the effect is wholly mediated. In step (5), we compare whether ω_2_×ω_4_ and ω_3_ are the same, if they are the same, indicating a partial mediating effect, if different, indicating the presence of a masking effect. The above process can determine whether the APPCAP policy is associated with sulfur dioxide intensity by influencing the mediating variable, total factor productivity (TFP).

#### 3.1.1. Measures of sulfur dioxide intensity

Sulfur dioxide emission and GDP are selected to measure the magnitude of sulfur dioxide intensity in our work, and we use sulfur dioxide emissions to GDP to express sulfur dioxide intensity. In particular, our empirical analysis below is dominated by Log (sulfur dioxide intensity) and supplemented by sulfur dioxide intensity.

#### 3.1.2. Measures of the APPCAP policy

*Time* × *Treat*, our core explanatory variable, represents a proxy measure for the APPCAP policy shock. Specifically, if a city is an APPCAP city, then the key explanatory variable is 1 in the ongoing and subsequent years and 0 in all others. There are 47 cities in the “three districts and ten groups” that belong to the vital air pollution areas in APPCAP policy, such as Beijing–Tianjin–Hebei, Yangtze River Delta, Pearl River Delta, central Liaoning, Shandong, Wuhan, and its surrounding areas, Chang-Zhu-Tan, Chengdu, Chongqing, the economic zone on the west side of the straits, central and northern Shanxi, Shaanxi, Guanzhong, Ganning and Urumqi, accounting for 17.34% of all-city sample (271 cities) during sample period. These 47 cities are treated as the pilot cities (i.e., treated groups), and the rest 224 cities are treated as the non-pilot cities (i.e., control groups). The mean values of the critical variables in the pilot and non-pilot cities are provided in Table 1.

**Table 1:** The main values of key variables in the pilot and non-pilot cities.

**Variables**	**Non-pilot period**		**Pilot period**	
	**Non-pilot samples**	**Pilot samples**	**Non-pilot samples**	**Pilot samples**
Sulfur dioxide intensity	109.748	46.728	51.687	16.192
Urbanization level	51.812	52.952	52.760	52.559
Proportion of secondary industry	51.155	51.735	46.496	45.980
Proportion of tertiary industry	34.355	41.183	40.783	48.939
Industrialization level	44.646	44.665	41.424	41.710
Per capita gdp (yuan)	63,626.530	109,657.200	57,801.030	114,894.700
Proportion of energy saving expenditure	3.098	2.781	55,862.660	24,312.610
Population density (10,000 people/km^2^)	386.577	803.607	390.729	769.853
Energy consumption (10,000 tons of coal)	1,298.165	3,120.640	970.167	2,347.052
FDI (10,000 yuan)	45,117.020	281,775.100	57,811.600	320,500.500
Rainfall (mm)	959.056	1,082.276	3,740.900	4,717.751

#### 3.1.3. Measures of TFP

Malmquist was first proposed by the Swedish economist Sten Malmquist in 1953 and has been widely recognized by the academic community. Therefore, this paper uses Malmquist to measure TFP, and its calculation formula is as follows:


(5) $$\begin{array}{l} Malmquist_{t}=\frac{D^{t}\left(x^{t+1},y^{t+1}\right)}{D^{t}\left(x^{t},y^{t}\right)} \end{array}$$

#### 3.1.4. Source of data

APPCAP has been implemented since 2013, and therefore, this study selects 2008–2018 as the regression period. The data of the explained and explanatory variables and control variables in our study are obtained from the “China City Statistical Yearbook,” “China Statistical Yearbook on the Environment from 2008 to 2018,” and “The local Statistical Yearbook.” The per capita GDP is deflated using the per capita GDP index based on 1978. Foreign direct investment (FDI) is initially converted using the exchange rate and then deflated using the fixed asset investment price index in 1990 as the base year. The descriptive statistics of the variables are listed in Table 2.

**Table 2:** Descriptive statistics.

**Variables**	**Unit**	**Obs**	**Mean**	**Std. dev**.	**Min**	**Max**
Sulfur dioxide intensity	Tons/100 million yuan	2,663	69.82	98.069	0.078	1,117.189
Per capita GDP	Yuan	2,758	69,588.108	44,479.502	11,282.535	704,725.910
Industrial structure	Secondary/tertiary	2,758	1.358	0.597	0.234	5.714
Industrialization level		2,393	42.957	12.015	3.91	87.252
Population density	Person/km^2^	2,733	457.602	390.154	1.499	3,822.741
Foreign investment	10,000 yuan	2,621	98,399.062	202,774.81	17	3,175,038.7
Rainfall	Annual average rainfall	2,763	2,595.635	3,860.889	29.3	20,855.373

## 4. Empirical findings

### 4.1. Baseline results

Table 3 reveals the baseline regression results of the effect of China's APPCAP policy on sulfur dioxide intensity. The logarithm of sulfur dioxide intensity is taken as the outcome variable. Both columns (1) and (2) control for the city-fixed effects, whereas the difference is that column (2) also adds six city-level control variables. Furthermore, we include year-fixed effects; the results are in columns (3) and (4). Whether control variables are included or not, the coefficients of *Time*×*Treat* are significantly negative at the level of 1%, indicating that the APPCAP policy can reduce the sulfur dioxide intensity in pilot cities.

**Table 3:** DID regression results.

**Variables**	**(1)**	**(2)**	**(3)**	**(4)**
	**Sulfur dioxide** **intensity (ln)**	**Sulfur dioxide** **intensity (ln)**	**Sulfur dioxide** **intensity (ln)**	**Sulfur dioxide** **intensity (ln)**
Time^*^treat	−1.273^***^	−0.630^***^	−0.243^***^	−0.230^***^
	(−15.860)	(−11.343)	(−5.261)	(−4.928)
Per capita GDP (ln)		0.211^***^		0.328^***^
		(3.361)		(6.744)
Industrial structure		0.471^***^		−0.032
		(10.319)		(−0.820)
Industrialization level (%)		0.133		0.010
		(0.519)		(0.050)
Population density (ln)		−0.011		0.018
		(−0.163)		(0.345)
FDI (ln)		−0.102^***^		−0.011
		(−5.060)		(−0.676)
Rainfall (ln)		−0.559^***^		−0.084^***^
		(−34.086)		(−3.358)
City fixed effect	√	√	√	√
Year fixed effect			√	√
Constant term	3.664^***^	5.714^***^	4.372^***^	1.341^**^
	(199.354)	(7.096)	(149.657)	(2.087)
N	2,663	2,209	2,663	2,209
R^2^	0.095	0.544	0.754	0.731

t statistics in parentheses,^*^p < 0.1, ^**^p < 0.05, ^***^p < 0.01.

Through the analysis of column (4) in Table 3, we can find that the estimated coefficient of the core explanatory variable is reduced by 0.230 due to the implementation of the APPCAP policy. In other words, sulfur dioxide intensity in pilot cities dropped by an average of 23% compared to non-pilot cities. Furthermore, the APPCAP policy was first implemented in 2013; hence, the DID estimate captures a 6-year average treatment effect. This means the APPCAP cities reduce the intensity of SO2by 3.83% per year (23%/6). In sum, China's APPCAP policy effect is statistically and economically significant.

Considering the control variables, we primarily figure out the estimation results in column (4) of Table 3. The coefficient of GDP per capita is statistically and significantly negative, implying that the increase in GDP per capita comes at the cost of more air pollution. The positive effect of GDP per capita on sulfur dioxide intensity outweighs the negative effects. The annual average rainfall is significantly and negatively associated with sulfur dioxide intensity, illustrating that cities with higher rainfall can effectively remove SO_2_, in line with the findings of Dong et al. (23). As presented in Table 3, other control variables, such as industrialization level and population density, do not remarkably affect sulfur dioxide intensity.

### 4.2. Dynamic DID

**Figure 4:**
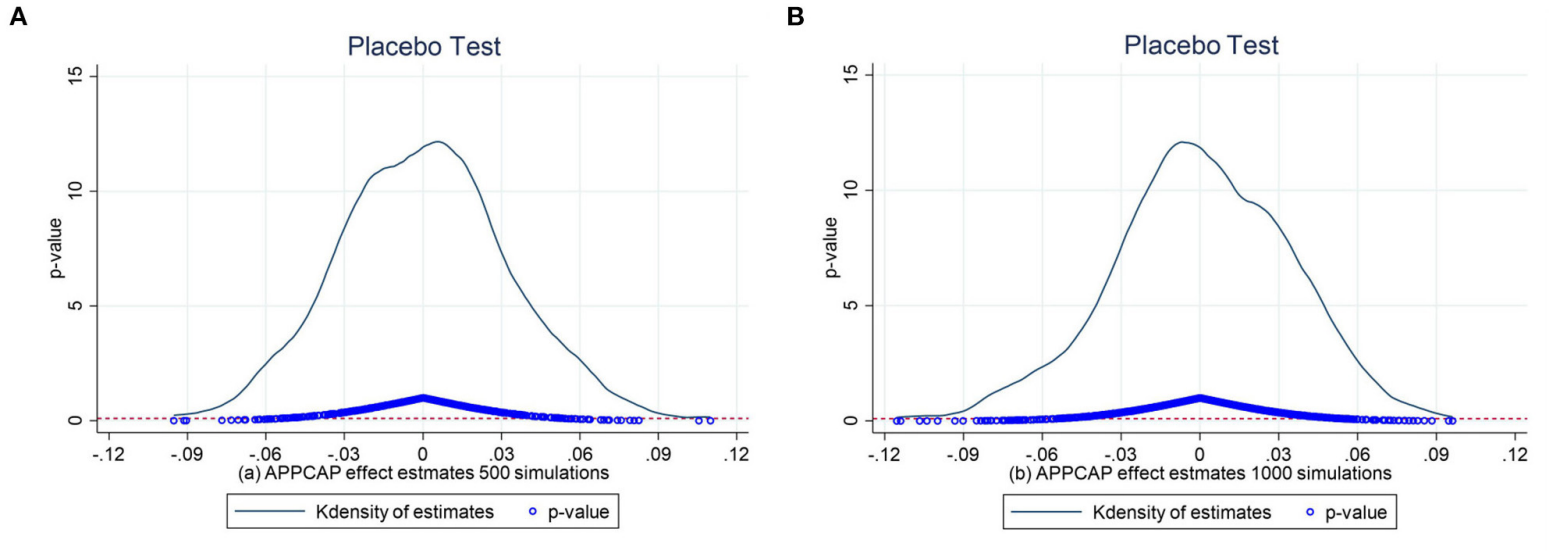
Placebo test. **(A)** 500 times. **(B)** 1,000 times.

**Figure 5:**
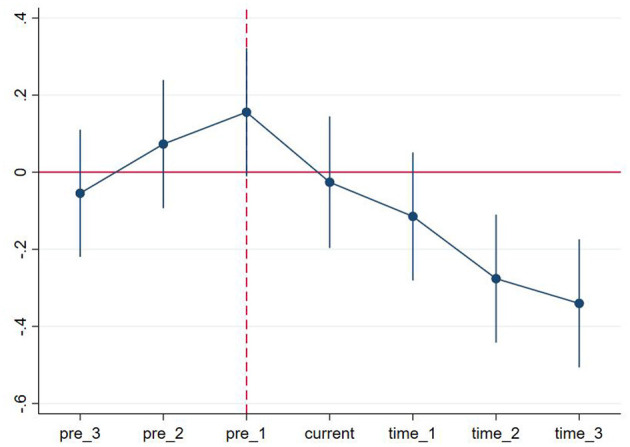
Parallel trend test of sulfur dioxide intensity in the experimental and control groups.

The DID model requires no systematic differences in the trend of sulfur dioxide intensity before implementing the APPCAP policy, and even if there are differences, these differences are fixed. The horizontal axis in Figure 4 implies the years before and after the APPCAP policy, e.g., pre_3 suggests the third year prior to the APPCAP policy and time_3 presents the third year after the APPCAP policy. The results of the parallel trend test shown in Figure 5 show that the coefficient estimates for the APPCAP pilot cities in the periods leading up to policy implementation are insignificant. In the second year after the implementation of the policy, the estimation coefficient gradually becomes significant and negative, which indicates that the implementation of the policy has a specific lagging effect and cannot have significant results in the current period of policy issuance. In summary, there was no significant difference between the pilot and non-pilot cities before the policy implementation, and the effect began to be produced in the second year after the policy implementation, so we consider that our study sample passed the parallel trend test.

### 4.3. Robustness checks

#### 4.3.1. PSM-DID estimates

In order to solve systematic differences, we further combine propensity score matching with DID (PSM-DID). Namely, we cannot guarantee that APPCAP and non-APPCAP cities have similar characteristics before implementing the APPCAP policy. There may be a considerable variation in the 271 cities we have chosen as samples before, so we use the PSM-DID approach to mitigate systematically. Table 4 shows the PSM-DID test results, indicating that the APPCAP can reduce the sulfur dioxide intensity because the four cross-term coefficients are significant at the significant level of 1%. The PSM-DID approach is the same as the results of the DID approach, and there is no significant difference in the estimated coefficients of the interaction, implying that the APPCAP policy still effectively reduces sulfur dioxide intensity, lending further support to the robustness of the results.

**Table 4:** PSM-DID estimates.

**Variables**	**(1)**	**(2)**	**(3)**	**(4)**
	**Sulfur dioxide** **intensity (ln)**	**Sulfur dioxide** **intensity (ln)**	**Sulfur dioxide** **intensity (ln)**	**Sulfur dioxide** **intensity (ln)**
Time^*^treat	−1.273^***^	−0.630^***^	−0.243^***^	−0.230^***^
	(−15.860)	(−11.343)	(−5.261)	(−4.928)
Control variables		√		√
City fixed effect	√	√	√	√
Year fixed effect			√	√
Constant term	3.664^***^	5.714^***^	4.372^***^	1.341^**^
	(199.354)	(7.096)	(149.657)	(2.087)
N	2,663	2,209	2,663	2,209
R^2^	0.095	0.544	0.754	0.731

t statistics in parentheses, ^*^p < 0.1, ^**^p < 0.05, ^***^p < 0.01.

#### 4.3.2. Placebo test

To check whether omitted variables influence the DID estimates, we designed a placebo test by randomly assigning APPCAP status to cities so that we can figure out whether our findings are the result of APPCAP policy. Because 47 cities are treated as pilot cities, we decided to randomly select the same number of cities as the treatment group, constituting a pseudo-interaction term. Then, we perform the regression with the setting of Equation (1). Based on our assumptions, and the results should not be remarkable unless the random cities are the same as the actual cities. To make the results more convincing, we ran the random procedure 500 and 1000 times, respectively.

Figures 4A, B show the distribution of the *p*-value obtained from 500 and 1,000 simulations randomly assigning the APPCAP status to cities and the curve is the kernel density distribution of the estimation coefficient. Furthermore, the horizontal dashed line is a significance level of 0.1, and most of the estimates' *p*-value is more prominent than 0.1, implying that passing the great test is a small probability event. These observations suggest that unobserved factors do not drive the adverse and significant effects of APPCAP on sulfur dioxide intensity.

#### 4.3.3. Substitute dependent variable

To further eliminate the possibility of primary regression results being influenced by other factors, we conduct a robustness test on the DID model result by substituting the dependent variable because we consider that the impact of the APPCAP is not limited to sulfur dioxide intensity. Therefore, we choose carbon dioxide emissions and carbon intensity as the dependent variable. We can assume that the result is robust if the cross-term coefficients between the time and treat dummy variable are remarkable. The result in Table 5 shows that the core conclusions of this study still stand.

**Table 5:** Substitute dependent variable estimates.

**Variables**	**(1)**	**(2)**	**(3)**	**(4)**
	**Carbon dioxide (ln)**	**Carbon dioxide (ln)**	**Carbon intensity (ln)**	**Carbon intensity (ln)**
Time^*^treat	−0.053^***^	−0.051^***^	−0.003^**^	−0.001^*^
	(−7.253)	(−7.159)	(−2.423)	(−1.671)
Control variables		√		√
City fixed effect	√	√	√	√
Year fixed effect	√	√	√	√
Constant term	2.955^***^	2.474^***^	0.026^***^	0.057^***^
	(635.583)	(25.897)	(30.957)	(5.477)
N	2,723	2,241	2,722	2,241
R^2^	0.697	0.732	0.269	0.406

t statistics in parentheses, ^*^p < 0.1, ^**^p < 0.05, ^***^p < 0.01.

#### 4.3.4. DDD estimates

To further demonstrate that our results are robust, this study expands the DID model to a DDD model by introducing the dummy variable of the carbon emission trading scheme, and the areas covered by the carbon emission trading scheme include Chongqing, Beijing, Tianjin, Shanghai, Guangdong, and Hubei. The triple cross-term can be understood as a change in sulfur dioxide intensity of the pilot area after the implementation of the carbon emission trading scheme and the APPCAP simultaneously. The coefficient of triple cross-term can be understood as the net effect of APPCAP on sulfur dioxide intensity in pilot areas after taking into account initial differences in policy effect caused by various external factors, the influence of different explanatory variables, differences between pilot and non-pilot areas, and the implementation of carbon market construction policies. As we can see in Table 6, both columns (1) and (2) control for the city-fixed effects, whereas the difference is that column (2) also adds six city-level control variables. Furthermore, we include year-fixed effects; the results are given in columns (3) and (4). In column (4) of Table 6, the coefficients are significantly negative at the 5% level, indicating that the implementation of the APPCAP has significantly reduced the sulfur dioxide intensity in the pilot areas when the carbon emission trading scheme is implemented.

**Table 6:** DDD test estimates.

**Variables**	**(1)**	**(2)**	**(3)**	**(4)**
	**Sulfur dioxide** **intensity (ln)**	**Sulfur dioxide** **intensity (ln)**	**Sulfur dioxide** **intensity (ln)**	**Sulfur dioxide** **intensity (ln)**
Time^*^treat^*^market	0.814^***^	0.218	−0.238^**^	−0.234^**^
	(3.855)	(1.498)	(−2.081)	(−2.020)
Treat^*^carbon market	0.425	0.622^**^	0.892^***^	0.697^***^
	(1.029)	(2.288)	(4.048)	(3.281)
Time^*^carbon market	−1.106^***^	−0.556^***^	−0.078	−0.009
	(−10.556)	(−7.635)	(−1.311)	(−0.150)
Time^*^treat	−1.206^***^	−0.576^***^	−0.196^***^	−0.194^***^
	(−13.104)	(−9.179)	(−3.681)	(−3.649)
Control variables		√		√
City fixed effect	√	√	√	√
Year fixed effect			√	√
Constant	3.699^***^	5.926^***^	4.331^***^	1.464^**^
	(141.209)	(7.464)	(141.067)	(2.276)
*N*	2,663	2,209	2,663	2,209
*R* ^2^	0.136	0.559	0.756	0.733

t statistics in parentheses, ^*^p < 0.1, ^**^p < 0.05, ^***^p < 0.01.

### 4.4. IV estimates

Various potential factors can affect the selection of APPCAP cities, which will further lead to the estimation bias of the DID model. Furthermore, our DID estimates would inevitably suffer from endogeneity problems if these factors simultaneously influence *Time* × *Treat* and sulfur dioxide intensity. Therefore, our study takes an IV test as an additional robustness test by using the ventilation coefficient as an instrumental variable based on Hering and Poncet (24).

An IV should satisfy both relevance and exogenous. First, does the ventilation coefficient satisfy the relevance requirement? According to the study by Box (25), wind speed influences horizontal pollution dispersion, and the mixed layer height can affect vertical pollution dispersion. Therefore, we define the ventilation coefficient as the product of wind speed and mixed layer height. The higher the value, the faster the pollution spreads; this means that there is a negative relevance between the ventilation coefficient and the sulfur dioxide intensity, in line with the relevance requirement. Second, does the ventilation coefficient satisfy the exogenous requirement? According to the method of Yu et al. (7), we collect wind speed information for 10 m height and boundary layer height (used to measure mixed layer height for 75 × 75 grids) in the ERA data. This data is from the European Center for Medium Weather Forecasting (ECMWF), which means that the ventilation coefficient has no direct relationship with sulfur dioxide intensity. Therefore, the ventilation coefficient fulfills the exogenous requirement.

Table 7 represents the estimated results of our IV test, and the estimated coefficients of Time^*^ventilation coefficient from column (1) to column (4) are significantly negative at the 1% level. Therefore, our results are still robust. To sum up, even considering the potential selection bias problem in pilot cities, APPCAP policy still contributes to reducing sulfur dioxide intensity, which highlights the robustness of the core finding of this study.

**Table 7:** IV estimates.

**Variables**	**(1)**	**(2)**	**(3)**	**(4)**
	**Sulfur dioxide** **intensity (ln)**	**Sulfur dioxide** **intensity (ln)**	**Sulfur dioxide** **intensity (ln)**	**Sulfur dioxide** **intensity (ln)**
Time^*^ventilation coefficient	−3.060^***^	−1.464^***^	−4.133^***^	−2.687^***^
	(−4.594)	(−2.950)	(−10.893)	(−7.733)
Control variables		√		√
Year fixed effect	√	√		
City fixed effect			√	√
Constant term	4.355^***^	1.168	3.931^***^	3.587^***^
	(35.164)	(1.507)	(94.768)	(3.232)
N	2,653	2,200	2,653	2,200

z-statistics in parentheses, ^*^p < 0.1, ^**^p < 0.05, ^***^p < 0.01.

### 4.5. Heterogeneity analysis

The pilot areas differ in many ways, for instance, in the climate, human factors, economic conditions, and natural environment. Therefore, to test whether regional differences will lead to bias in the regression results, in other words, we need to figure out if the APPCAP policy affects the pilot areas in different conditions, so we perform a heterogeneity analysis. We further divide the 47 pilot cities into three dimensions for DID regression, respectively, and the three dimensions are east and mid-west, heating and non-heating, and coastal and inland. Finally, we can consider our conclusion of this study to be robust if the cross-term coefficient is remarkable. Table 8 shows that all the cross-term coefficients are significantly negative at the 1% level, which means that the APPCAP policy has significantly reduced the sulfur dioxide intensity in the east or mid-west, heating or non-heating, and coastal and inland pilot areas.

**Table 8:** Heterogeneity analysis.

**Variables**	**(1)**	**(2)**	**(3)**	**(4)**	**(5)**	**(6)**
	**Sulfur dioxide intensity (ln)**	**Sulfur dioxide intensity (ln)**	**Sulfur dioxide intensity (ln)**	**Sulfur dioxide intensity (ln)**	**Sulfur dioxide intensity (ln)**	**Sulfur dioxide intensity (ln)**
Time^*^treat	−0.200^***^	−0.416^***^	−0.176^***^	−0.255^***^	−0.285^***^	−0.240^***^
	(−3.294)	(−4.805)	(−2.621)	(−3.859)	(−3.202)	(−4.193)
Control variables	√	√	√	√	√	√
City fixed effect	√	√	√	√	√	√
Year fixed effect	√	√	√	√	√	√
Constant term	3.043^***^	−1.754^*^	1.123	1.117	1.270	0.620
	(3.325)	(−1.865)	(1.139)	(1.255)	(0.959)	(0.815)
N	1,031	1,178	1,083	1,126	514	1,695
R^2^	0.732	0.749	0.742	0.731	0.726	0.741

t statistics in parentheses; ^*^p < 0.1, ^**^p < 0.05, ^***^p < 0.01.

### 4.6. Impact mechanism test

Table 9 reports the regression results for Equation (2), (3), and (4). Column (1) is the result of regression to Equation (1); the test results in column (1) show that the influence coefficient of the core explanatory variables on sulfur dioxide intensity is −0.299, which is significantly negative at the 1% level. Furthermore, it means that the implementation of the APPCAP policy can reduce the sulfur dioxide intensity. Column (2) is the result of regression to Equation (2), showing that the coefficient is insignificant. Column (3) is the result of regression to Equation (3). The test results in column (3) show that after controlling TFP, the impact coefficient of the core explanatory variables on sulfur dioxide intensity is −0.289, which is significantly negative at the 1% level. So we cannot directly conclude whether TFP has a mediating effect, so we further perform a Bootstrap test to find out whether the mediating effect of TFP exists. Based on the above results, we further perform a Bootstrap test, as shown in Table 10, the revised confidence interval does not contain 0, so we can conclude that implementing the APPCAP policy can reduce the sulfur dioxide intensity, and TFP failed to play a mediating role in it.

**Table 9:** Impact mechanism estimates.

**Variables**	**(1)**	**(2)**	**(3)**
	**Sulfur dioxide intensity (ln)**	**TFP**	**Sulfur dioxide intensity (ln)**
Time^*^treat	−0.299^***^	0.068	−0.289^***^
	(−4.576)	(0.947)	(−4.456)
TFP			−0.113^***^
			(−5.969)
Control variables	√	√	√
City fixed effect	√	√	√
Year fixed effect	√	√	√
N	2,164	2,204	2,164
R^2^	0.325	0.001	0.336

Standard errors in parentheses, ^*^p < 0.1, ^**^p < 0.05, ^***^p < 0.01.

**Table 10:** Bootstrap test: Total factor productivity.

**Trails**	**Effect**	**ω-value**	**S**	**Z-value**	***P*-value**	**95% confidence interval**	
						**Lower limit**	**Upper bound**
ω_3_	Direct effect	−0.235	0.035	−6.66	0.000	−0.304	−0.180
ω_2_^*^ω_4_	Intermediary effect	0.001	0.001	0.99	0.321	0.001	0.003

## 5. Conclusions and policy implications

It is generally believed that implementing environmental regulatory policies is an effective tool for air pollution governance. To improve air quality, China has piloted the APPCAP policy since 2013. Evaluating the influence of the APPCAP policy on sulfur dioxide intensity is of great significance for environmental regulatory policy formulation and economic development in China and other developing countries. Based on a panel data covering 271 prefecture-level cities between 2008 and 2018, our paper isolates the effect of the APPCAP policy on sulfur dioxide intensity with the employment of DID method and PSM-DID approach. We draw several main findings: (1) The baseline results suggest a 23% reduction in sulfur dioxide intensity in APPCAP cities compared to non-APPCAP cities. (2) An IV test deals with potential endogeneity problems, corroborating the baseline results. (3) The results of the DDD model suggest that China's APPCAP still exerts significant adverse effects on sulfur dioxide intensity in the pilot areas of the carbon emission trading scheme. (4) Our mechanism analysis indicates that total factor productivity (TFP) fails to play a partial mediating role in reducing the sulfur dioxide intensity under the implementation of the APPCAP policy. (5) The APPCAP policy significantly adversely affects the sulfur dioxide intensity in the heterogeneous analysis.

We derive three policy recommendations from our results. First, the Chinese government should further expand the scale of its APPCAP policy. Our main finding is that the APPCAP policy has a powerful sulfur dioxide intensity reduction effect. Further expansion of the APPCAP policy can improve air quality while ensuring rapid economic development. Moreover, non-APPCAP regions can learn from APPCAP regions' successful experiences. Because of the regional difference, the APPCAP policy should be formulated with sufficient consideration of local air pollution and economic development. It is vital to combine policies tailored to local conditions. In addition, if we properly combine the APPCAP policy with other local policies in different cities, it will likely have an excellent synergistic effect.

Second, the government should further improve the APPCAP policy to cover as many air pollutants as possible and specify specific emission control targets, such as adding long-term specific emission control indicators for air pollutants such as SO_2_, NO_2_, CO, and O_3_, in order to achieve the vision of gradually upgrading air quality standards and catching up with national air quality standards as soon as possible. At the same time, the specific formulation of policy formulation should also depend on improving air quality and cannot be rigid, blind, and “one-size-fits-all.”

Third, as a potential intermediary factor, total factor productivity is vital in reducing sulfur dioxide intensity under the APPCAP policy, while the effectiveness of it is poor in this study. Therefore, China's local government departments should pay more attention to the energy efficiency of enterprises and how to improve the energy efficiency of enterprises in the first place, which not only has a positive impact on air governance but also helps to improve the level of local economic development. In addition, government departments should establish the concept of green development and innovative development, increase investment in R&D subsidies for local enterprises, and encourage enterprises to develop new technologies and improve energy efficiency to improve air quality.

## Data availability statement

The original contributions presented in the study are included in the article/supplementary material, further inquiries can be directed to the corresponding author.

## Author contributions

SN and YF: conceptualization and visualization. SN: methodology and software. RZ: validation and funding acquisition. YF: formal analysis. YC and YF: data curation, writing–review and editing, and supervision. YC: writing–original draft preparation. All authors have read and agreed to the published version of the manuscript.
